# Transcription Accuracy of Automatic Speech Recognition for Orthodontic Clinical Records

**DOI:** 10.1177/00220345251382452

**Published:** 2025-11-03

**Authors:** R. O’Kane, D. Stonehouse-Smith, L.C.U. Ota, R. Patel, N. Johnson, C. Slipper, J. Seehra, S.N. Papageorgiou, M.T. Cobourne

**Affiliations:** 1 Centre for Craniofacial Development & Regeneration, Faculty of Dentistry, Oral & Craniofacial Sciences, King’s College London, London, UK; 2 Department of Orthodontics, Guy’s and St Thomas’ NHS Foundation Trust, London, UK; 3 Department of Orthodontics, Royal Alexandra Children’s Hospital, Brighton and Sussex University Hospitals NHS Foundation Trust, Sussex, UK; 4 Clinic of Orthodontics and Pediatric Dentistry, Center of Dental Medicine, University of Zurich, Switzerland

**Keywords:** artificial intelligence, dentistry, electronic health records, large language model, natural language processing, speech recognition software

## Abstract

Accurate clinical records are fundamental to dental practice. Automatic speech recognition (ASR) has the capacity to convert spoken clinical language into written text within the electronic health record; however, the accuracy of ASR in natural language processing for clinical dentistry remains uncertain. The aim of this study was to investigate the transcriptional accuracy of ASR systems using orthodontic clinical records as the experimental model. Specifically, we used 4 commercial ASR systems (Heidi Health, DigitalTCO, Dragon Medical One, Dragon Professional Anywhere), 5 application programming interfaces (Amazon, Google, Speechmatics, Whisper, GPT4oTranscribe), and a 2-stage pipeline coupling GPT4oTranscribe with the GPT4o large language model (LLM) for generative error correction (GPT4oTranscribeCorrected). Orthodontic diagnostic and treatment planning summaries (*n* = 200; 10 subject domains; 43,408 words; 6 h of audio) were narrated and recorded for analysis. The primary outcome was domain word error rate (DWER), which investigates clinical terminological transcription errors against the Systematized Nomenclature of Medicine Clinical Terms (SNOMED-CT) database. Secondary outcomes included nondomain WER (N-DWER), lexical accuracy (Recall-Oriented Understudy for Gisting Evaluation [ROUGE] score), semantic similarity (Bidirectional Encoder Representations from Transformers [BERT] and Bidirectional and Auto-Regressive Transformer [BART] scores), hallucinations (transcribed text not in the spoken input), and qualitative error analysis. GPT4oTranscribeCorrected was transcriptionally most accurate (DWER = 3.5%; WER = 3.7%), with DWER decreasing by 54.9% versus GPT4oTranscribe. Heidi Health was the highest-performing commercial system (DWER = 6.2%; WER = 5.4%), with Dragon Professional Anywhere being the worst (WER = 33.9%). All systems were less accurate with technical vocabulary (DWER > N-DWER; *P* < 0.001), except GPT4oTranscribeCorrected. Significant differences were seen across systems for ROUGE, BERT, and BART scores (*P* < 0.001). Based on post hoc pairwise comparisons, GPT4oTranscribeCorrected performed best and Dragon Professional Anywhere was consistently worst for lexical and semantic errors. Hallucinations were absent except for Whisper (*n* = 57) and DigitalTCO (*n* = 1). Across systems, background noise increased DWER and WER (*P* < 0.001). Importantly, clinically significant errors were seen with all systems, ranging from 2% to 66% (GPT4oTranscribeCorrected clean; Dragon Medical One background noise, respectively). Variation in narrator accent had no effect in clean conditions (*P* = 0.65) and a small effect with background noise (*P* = 0.001). ASR systems deliver single-digit transcription error rates, particularly when coupled with LLM-based correction, but clinically significant errors persist. The verification of clinical records is essential when using current ASR systems.

## Introduction

Accurate and contemporaneous patient records are fundamental to health care and essential for documenting diagnosis, treatment, and continuity of care. In the United Kingdom, record keeping in dentistry is guided by established standards from professional bodies and specialist societies (General Dental Council 2013; British Orthodontic Society 2022).

Electronic health records (EHRs) (see Appendix Table 1 for a full list of acronyms) are increasingly used by health care providers to collate and store patient data within and between organizations (Heart et al. 2017). These data include patient demographics, medical history, diagnostics, charting, treatment planning and process, and clinical outcomes, often extending across multiple specialties. EHRs offer significant operational advantages compared with analog systems, including clear, accurate, and accessible patient documentation; seamless connectivity across platforms; template commonality; and coding functionality. These features can help organizations with workflow optimization, regulatory compliance, data accessibility for research and audit, cloud-based storage, and increased accessibility to medical records. Moreover, patient referral, appointment scheduling, and contract management can all be managed electronically (Honavar 2020).

Despite considerable advantages associated with EHRs, there are also challenges, including institutional implementation, security risks, and issues with data-input reliability, often perpetuated through the extensive operator use of “copy and paste” (Ozair et al. 2015; Falcetta et al. 2023). Indeed, a key component of EHR functionality is the reliance on data input directly by clinicians during the patient consultation (Boonstra et al. 2022). This can negatively effect patient–clinician interactions, reducing eye contact, increasing clinician response times while computer-based tasks are completed (introducing pauses and encouraging distracting patient questions), and fostering a consultation environment where technology becomes the dominant presence (Crampton et al. 2016; Marino et al. 2023). To overcome some of these disadvantages, in-office or remote human scribes have been trialed to supplement the EHR, recording patient information and clinical notes during consultations, allowing clinicians to focus directly on the patient. These interventions can reduce documentation burden, improve efficiency, and increase work satisfaction, but they require additional manpower in the form of a scribe (Gidwani et al. 2017; Mishra et al. 2018; Micek et al. 2022).

Automatic speech recognition (ASR) is the process in which a machine recognizes spoken human language and transcribes these data into written text (O’Shaughnessy 2024). A key application for ASR in health care is the generation of clinical documentation, including transcription of clinical notes, letters, and doctor–patient consultations. There is evidence from pilot studies that within these contexts, ASR can be convenient, accurate, and expeditious (Latif et al. 2021). Nonetheless, challenges remain, particularly with clinical and technical terminology, which can be associated with errors necessitating substantial posttranscription editing (Quiroz et al. 2019). In particular, mistranscriptions are recognition errors in output that distort spoken words, whereas hallucinations invent or contradict information not found in the original spoken text (Ji et al. 2023). Indeed, developing robust ASR tailored to specialist subjects such as dentistry is often complicated by the difficulty and expense of acquiring the large volumes of accurately transcribed, domain-specific training data. However, recent developments in deep neural networks and machine learning have brought improvements in ASR capabilities, including tools that can leverage natural language processing and large language models (LLMs) (Xiong et al. 2017; Israni and Verghese 2019). One particularly promising approach is generative error correction (GEC), which uses an LLM to automatically identify and correct ASR errors (Errattahi et al. 2018; Ma et al. 2023).

Many contemporary systems use an ASR speech-to-text application programming interface (API) available from multiple multinational-technology companies. These newer tools should be more adept at understanding context and specialized domain-specific terminology, with recent research demonstrating the efficiency of these ASR systems in medicine (Sezgin et al. 2023; Liu et al. 2024). Given these findings, and the growing availability of this technology in clinical dentistry, we conducted a pilot study investigating the timing of dental clinical summary generation using narration with an ASR-LLM versus manual typing, identifying time reductions of almost 60% with ASR (Appendix Table 2). The aim of the present study was to investigate the transcriptional accuracy of ASR systems in dentistry using narrated orthodontic clinical records, specifically, transcriptional, lexical, and semantic accuracy using validated metrics and qualitative error analysis, including hallucinations and mistranscriptions.

## Materials and Methods

Ethical approval was granted by King’s College London as a minimal risk investigation (MRA-24/25-46684). This study adheres to the World Health Organization–International Telecommunication Union checklist for artificial intelligence (AI) research in dentistry (Schwendicke et al. 2021). A detailed description of the materials and methods is available in the Appendix.

We evaluated 10 distinct ASR systems for orthodontic clinical record keeping with selection based on accessibility, functionality, and relevance to the international dental community. The first category represented directly available commercial systems (*n* = 4): Dragon Medical One and Dragon Professional Anywhere (Nuance Communications, Microsoft Corp; dictation products specifically trained for health care terminology or general purpose dictation, respectively) and ambient scribes designed for clinical use in dentistry: Heidi Health AI Digital Scribe (Heidi Health) and DigitalTCO (DigitalTCO). The second category represented standalone speech-to-text API systems, providing direct access to widely available, foundational state-of-the-art ASR models that underpin many downstream applications, allowing the direct evaluation of core-engine performance (*n* = 5): Speechmatics Enhanced (Speechmatics), GPT4oTranscribe (OpenAI), Whisper OpenAI Large version 2 (Whisper), Amazon Medical Transcribe (Amazon Web Services), and Google Medical (Google LLC). The third category represented an experimental ASR-LLM consisting of a 2-stage automated pipeline labeled GPT4oTranscribeCorrected, evaluating the impact of using a GPT4o LLM to perform GEC on GPT4oTranscribe ASR output (OpenAI) (Fig. 1). The processes of prompt construction, validation, and temperature for the GEC step is detailed in the Appendix (Appendix Tables 3 and 4).

**Figure 1.**
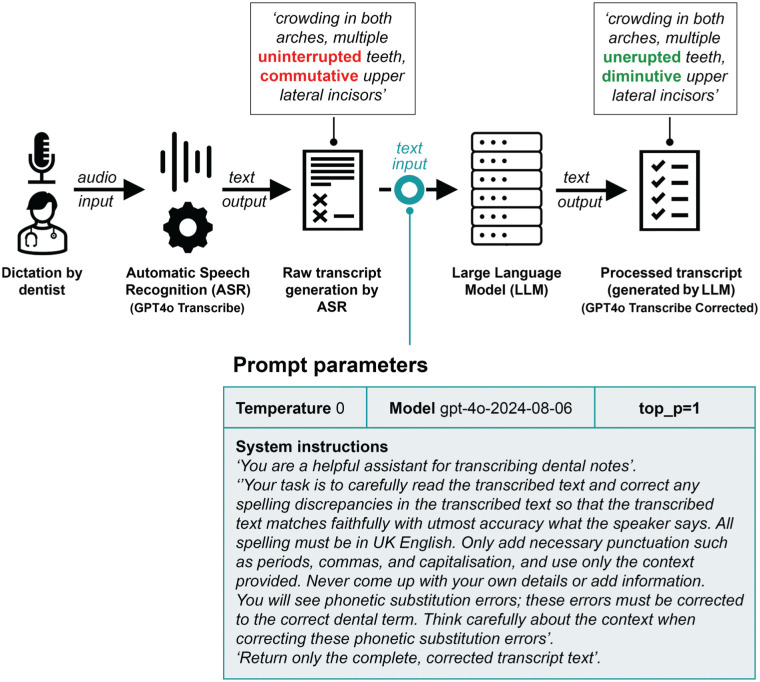
Schematic representation of the 2-stage transcription pipeline. Dictated audio is recorded, and the audio file is processed by an automatic speech recognition system (in this example, GPT4oTranscribe) to produce a raw text transcript. The raw text transcript is then passed to a large language model (LLM; GPT4o) together with the prompt shown (temperature 0, top_p=1). The LLM performs generative error correction to provide the final transcript. Misrecognized terms are highlighted in red (uninterrupted, commutative); corrected terms are highlighted in green (unerupted, diminutive). The figure example is the generated text from Transcript 20 (with background noise), GPT4oTranscribe (raw transcript), and GPT4oTranscribeCorrected (processed transcript).

We used orthodontic clinical records incorporating diagnosis and treatment planning as our experimental model because these cover a wide range of technical language relevant to dentistry, including (but not limited to) craniofacial anatomy and cephalometrics, genetics and craniofacial growth, anatomic tooth notation, dental disease, occlusal classification, clinical indices, treatment mechanics, appliance systems, and surgical interventions. Based on the sample size calculation (*n* = 200), transcripts were generated to provide a library of contemporary clinical records. Collectively, these transcripts were narrated to create the audio files and served as the ground truth for investigating each ASR system. In addition, all systems were evaluated in the presence or absence of background noise and a subset with variation in narrator accent.

Each system was assessed for transcriptional, lexical, and semantic accuracy using validated word and character error metrics. The primary outcome was domain word error rate (DWER), which assesses transcription accuracy involving clinical terminology. Error analysis also involved manual review to identify hallucinations and categorize mistranscriptions, focusing on the clinical significance of class 3 transcription errors, which alter clinical meaning and potentially affect patient care.

All primary data are available at Zenodo: https://doi.org/10.5281/zenodo.15470163.

## Results

Table 1 shows the median word and character transcription error metrics for each ASR system. Significant differences were seen for DWER, nondomain word error rate (N-DWER), word error rate (WER), unnormalized word error rate (uWER), and character error rate (CER) across systems (*P* < 0.001, in all instances). Based on post hoc pairwise comparisons (Appendix Table 5), GPT4oTranscribeCorrected performed significantly better than all other systems, whereas GPT4oTranscribe and Heidi Health were consistently ranked second and third best, respectively. In contrast, Dragon Professional Anywhere was ranked worst for all transcription error metrics and Dragon Medical One second worst.

**Table 1.** Comparative Word and Character Error Metrics by ASR System.

Tool	DWER (%) Median (IQR)	WER (%) Median (IQR)	N-DWER (%) Median (IQR)	uWER (%) Median (IQR)	CER (%) Median (IQR)
Amazon Medical Transcribe (API)	23.43 (14.42, 32.88)	12.33 (9.09, 17.07)	14.07 (10.14, 18.81)	24.33 (19.02, 29.85)	5.50 (3.49, 7.60)
DigitalTCO	12.13 (6.78, 20.00)	6.44 (4.32, 9.42)	7.01 (4.58, 10.00)	13.84 (10.28, 17.85)	2.64 (1.59, 3.86)
Dragon Medical One	29.94 (20.00, 38.97)	24.06 (19.55, 29.71)	26.19 (21.47, 32.26)	38.17 (33.52, 42.73)	11.82 (9.28, 15.52)
Dragon Professional Anywhere	48.74 (38.24, 58.03)	33.91 (27.90, 40.17)	37.21 (30.37, 43.22)	46.01 (40.46, 52.46)	17.11 (13.58, 21.00)
Google Medical (API)	23.37 (15.19, 34.45)	10.97 (8.49, 15.88)	11.23 (8.35, 16.17)	18.65 (14.16, 23.11)	5.23 (3.37, 7.60)
GPT4o Transcribe	7.69 (0, 15.38)	5.13 (3.04, 7.49)	5.32 (3.19, 8.37)	11.30 (7.62, 14.63)	1.71 (1.04, 3.00)
GPT4o Transcribe Corrected	3.47 (0, 9.09)	3.73 (2.10, 6.16)	3.90 (2.08. 5.98)	10.06 (6.36, 13.00)	1.31 (0.69, 2.48)
Heidi Health	6.19 (1.72, 14.29)	5.40 (3.50, 7.91)	5.45 (3.60, 8.73)	11.67 (8.75, 15.74)	2.14 (1.28, 3.64)
Speechmatics Enhanced (API)	15.47 (6.78, 25.00)	9.06 (6.14, 12.5)	9.92 (6.55, 13.56)	19.01 (14.87, 24.81)	3.49 (2.37, 5.18)
Whisper OpenAI Large version 2 (API)	16.11 (9.09, 25.00)	10.97 (6.58, 21.14)	11.35 (6.99, 21.75)	17.91 (12.41, 27.92)	5.30 (2.47, 13.65)
*P* value	<0.001^a^	<0.001^a^	<0.001^a^	<0.001^a^	<0.001^a^

API, application programming interface; ASR, automatic speech recognition; CER, character error rate; DWER, domain word error rate; IQR, interquartile range; N-DWER, nondomain word error rate; uWER, unnormalized word error rate; WER, word error rate.

a Wald-type test for overall differences from quantile regression

Table 2 shows that with the exception of GPT4oTranscribeCorrected, ASR systems had considerable difficulty in recognizing domain-specific words, as demonstrated by DWER scores significantly higher than N-DWER (*P* ≤ 0.001). According to the effect size of the difference between DWER and N-DWER, Heidi Health, Whisper, and Dragon Medical One were the best performers, whereas Google Medical, Amazon Medical Transcribe, and Dragon Professional Anywhere were the worst.

**Table 2.** Performance Variability between DWER versus N-DWER (Wilcoxon Signed-Rank Test).

Tool	*P* Value^a^	Effect Size
Amazon Medical Transcribe (API)	<0.001^a^	0.644
DigitalTCO	<0.001^a^	0.551
Dragon Medical One	<0.001^a^	0.234
Dragon Professional Anywhere	<0.001^a^	0.632
Google Medical (API)	<0.001^a^	0.727
GPT4o Transcribe	<0.001^a^	0.329
GPT4o Transcribe Corrected	0.08^a^	0.020
Heidi Health	0.002^a^	0.170
Speechmatics Enhanced (API)	<0.001^a^	0.466
Whisper OpenAI Large version 2 (API)	0.001^a^	0.175

API, application programming interface; DWER, domain word error rate; N-DWER, nondomain word error rate.

a Bonferroni adjusted.

Comparative summarization metrics relating to lexical and semantic accuracy are shown in Table 3 (median Recall-Oriented Understudy for Gisting Evaluation [ROUGE], Bidirectional Encoder Representations from Transformers [BERT], and Bidirectional and Auto-Regressive Transformer [BART] scores) and Appendix Table 6 (median ROUGE-1: unigrams; ROUGE-2: bigrams; ROUGE-L: LCS, longest common subsequence). The mean ROUGE scores are shown in Appendix Figure 1. Significant differences were seen across systems for all metrics (*P* < 0.001, in all instances). Based on post hoc pairwise comparisons (Appendix Table 7), GPT4oTranscribe Corrected was again the best performer for all metrics, followed by GPT4oTranscribe and Heidi Health. The only exception was BART score, for which Heidi Health performed slightly worse than GPT4oTranscribe. Dragon Professional Anywhere was consistently ranked worst for lexical and semantic error metrics, with Dragon Medical One second worst.

**Table 3.** Comparative Summarization Metrics for Lexical (ROUGE Score) and Semantic (BERT, BART Scores) Accuracy and Hallucinations by the ASR System.

Tool	ROUGE, Median (IQR)	BERT, Median (IQR)	BART, Median (IQR)	Hallucinations, n (%)
Amazon Medical Transcribe (API)	0.875 (0.837, 0.909)	0.894 (0.870, 0.923)	−1.285 (−1.533, −0.997)	0 (0%)
DigitalTCO	0.937 (0.910, 0.957)	0.943 (0.924, 0.960)	−0.766 (−1.004, −0.574)	1 (0.5%)
Dragon Medical One	0.775 (0.732, 0.816)	0.785 (0.761, 0.813)	−2.186 (−2.706, −1.816)	0 (0%)
Dragon Professional Anywhere	0.691 (0.635, 0.739)	0.730 (0.701, 0.762)	−3.350 (−3.919, −2.786)	0 (0%)
Google Medical (API)	0.887 (0.851, 0.916)	0.916 (0.889, 0.940)	−1.125 (−1.501, −0.930)	0 (0%)
GPT4o Transcribe	0.947 (0.922, 0.968)	0.964 (0.943, 0.978)	−0.591 (−0.786, −0.473)	0 (0%)
GPT4o Transcribe Corrected	0.960 (0.938, 0.976)	0.972 (0.959, 0.983)	−0.471 (−0.594, −0.383)	0 (0%)
Heidi Health	0.948 (0.927, 0.968)	0.959 (0.941, 0.973)	−0.611 (−0.780, −0.488)	0 (0%)
Speechmatics Enhanced (API)	0.903 (0.877, 0.932)	0.930 (0.905, 0.944)	−0.948 (−1.180, −0.742)	0 (0%)
Whisper OpenAI large version 2 (API)	0.904 (0.856, 0.936)	0.934 (0.897, 0.957)	−0.955 (−1.350, −0.730)	57 (28.5%)
*P* value	<0.001^a^	<0.001^a^	<0.001^a^	<0.001^b^

ASR, automatic speech recognition; BART, Bidirectional and Auto-Regressive Transformer; BERT, Bidirectional Encoder Representations from Transformers; IQR, interquartile range; ROUGE, Recall-Oriented Understudy for Gisting Evaluation.

a Wald-type test for overall differences from quantile regression.

b Wald-type test for overall differences from the generalized linear model for the binomial family.

Hallucinations were not seen across 8 of the tools but did occur with Whisper (*n* = 57) and DigitalTCO (*n* = 1) (see Table 3; Appendix Table 8).

Table 4 presents the overall domain term missed ratios (averaged across ASR systems) and illustrative examples of mistranscriptions. This analysis revealed wide-ranging performance on specific domain terms, with some proving challenging. These examples qualitatively underscore the quantitative DWER findings, demonstrating that domain-specific terminology poses a significant challenge for current ASR systems and leads to frequent, varied transcription errors, even for common clinical terms.

**Table 4.** Domain Terms, Overall Domain Term Missed Ratio, and Corresponding Mistranscriptions across ASR Systems.

Domain Term	Overall Domain Term Missed Ratio, %	Example of Mistranscriptions
Essix	97.5	Essex, S6
Dentigerous cyst	75	Denture disc, decent disc, detention cyst, dentulous disc, detentious cyst
ANB angle	82.5	A and B, AMP, ANP
Molar incisor hypomineralization	90	Molar incisor hyper mineralization, molar incisor condition
Mesially	75	Nasally, easily, measly
Hawley	80	Holly, hollow, holey
Alendronate	66.67	Alan John it, Alan Janet, non-dominant, allodonate
Palatally	62.75	politically, politely
Curve of Spee	65	curve speed, currently Spee, curvies 3
Buccally	53.33	Buckly, buckley, broccoli
Cephalometric	49.13	Capim meric, cattle metric, Affy metrix, pathomeric, kefalometric
Mesiodistal	58.75	Easily distal, piece of distal, media distal
Unerupted	44.29	Uninterrupted, unruptured
Skeletal	38.21	Seletto, sceledo, scleral
Labial	22.38	Label, labelly
Overjet	13.40	Overjit, overjowt
Maxillary	12.18	Axillary, auxiliary, papillary
Pogonion	80	Ponion
Proclination	41.07	Proclamation, procreation, inclination, protonation

ASR, automatic speech recognition.

The overall domain term missed ratio (%) is the proportion of times a term was mistranscribed across all ASR systems. A 100% overall missed ratio indicates all ASR systems failed to transcribe the term correctly.

A qualitative error analysis using Kanal’s typology showed class 0 formatting and class I minor nonmeaning grammatical change errors common across systems (Appendix Table 9). Class 3 errors altering meaning with potential clinical impact were also seen across systems, ranging from *n* = 4 and *n* = 10 (GPT4oTranscribeCorrected, clean and background noise, respectively) to *n* = 353 and *n* = 567 (Dragon Medical One, clean and background noise, respectively). As a proportion of total errors generated, these clinically significant errors constituted 0.21% to 4.15% (GPT4o TranscribeCorrected, clean; Dragon Medical One, background noise, respectively). The proportion of transcripts containing at least 1 class 3 error ranged from 2% to 66% (GPT4oTranscribeCorrected, clean; Dragon Medical One, background noise, respectively) (Appendix Table 10). Qualitative review revealed that these errors could lead to significant clinical misinterpretation (Appendix Table 11). Overall, GPT4oTranscribeCorrected demonstrated the lowest proportion of class 3 errors relative to total error count, with Dragon Medical One being the highest.

Applying normalization rules lowered the WER for all systems. The normalized median WER was consistently below uWER, with absolute reductions ranging from 6.17% to 14.11% for GPT 4oTranscribe and Dragon Medical One, respectively.

Across systems, background noise increased WER (+0.01 95% CI 0.01–0.02, *P* < 0.001) and DWER (+0.04, 95% CI 0.03–0.05; *P* < 0.001) relative to clean audio, and the noise-by-system interaction terms were significant for both (*P* < 0.001) (Appendix Table 12). Overall, background noise degraded performance, but magnitude was system dependent, with the 2 GPT4o variants and Heidi Health displaying the greatest resilience.

Speaker accent exerted only a minor influence. Using speaker CO as the baseline, neither speaker RO nor RP altered the median WER in clean recordings (both −0.01 pp; overall *P* = 0.65). Taken together, ASR system choice dominated performance, whereas accent introduced marginal changes detectable only with in the presence of background noise (Appendix Table 13).

## Discussion

This study provides a comprehensive evaluation of contemporary ASR systems within the context of dental clinical record keeping using orthodontic diagnosis and treatment planning as the experimental model. Our findings reveal significant variability in transcriptional accuracy across systems and underlying speech-to-text APIs. Although technological advances are evident and will continue, achieving consistent reliable transcription, particularly for domain-specific terminology of clinical relevance, remains challenging.

A primary goal of ASR in health care is to alleviate the burden associated with data input for EHR. This is the first study to investigate ASR systems in dentistry using validated metrics. WER is a common metric used to investigate ASR, with modern systems capable of scores (>2%) exceeding human transcription (around 5%) for general language (Amodei et al. 2016; Zhang et al. 2022). Defining an “acceptable” WER for transcribed clinical documentation is difficult, and we reveal wide-ranging performance across systems. Among commercial systems, Heidi Health was most accurate; however, the integrated architecture in our experimental ASR-LLM pipeline was superior. These findings confirm high-level transcriptional accuracy with ASR; however, with the exception of GPT4oTranscribeCorrected, DWER scores were significantly higher than N-DWER, emphasizing the difficulties these systems have with technical (in this study, clinical orthodontic) vocabulary. Therefore, even ASR systems achieving low WER require careful scrutiny of clinical terminology before their reliability for unreviewed use can be ensured. The domain term missed ratio analysis further illustrated this, revealing even common orthodontic terms to be problematic. Indeed, some clinical terms exhibited high missed ratios across systems, often being substituted with phonetically similar but incorrect words. Developing robust ASR systems requires large amounts of accurately transcribed training data, often difficult and expensive to obtain, especially for specialist areas such as dentistry. To address these limitations, GEC using LLM is a promising method for automatically identifying and correcting ASR errors (Errattahi et al. 2018). LLMs demonstrate high performance across various applications, suggesting suitability for this purpose and likely because they have effectively “read” vast amounts of text (including dental terminology) more extensively than an ASR engine has “heard” it in more-limited training audio. Supporting this, with effective prompting, a pretrained LLM can equal or even surpass domain-specific language models (Yang et al. 2023). In our experimental ASR-LLM pipeline, we observed that incorporating the LLM significantly improved transcription accuracy, reducing both N-DWER and DWER.

Standard metrics quantify transcription errors, but they do not capture quality dimensions for clinical documentation, including preservation of specific phrasing, logical flow, and accurate semantics (Ye-Yi et al. 2003). To gain a more holistic understanding of transcript fidelity, we measured lexical similarity (ROUGE) (Lin 2004), semantic coherence (BERT), and fluency (BART) (Yuan et al. 2021). GPT4oTranscribeCorrected and GPT4oTranscribe generally performed best across these metrics, largely mirroring WER and DWER rankings. This convergence strongly suggests that top-performing systems not only make fewer word-level errors but are also more successful at generating transcripts lexically closer to the original narration, better preserving intended clinical meaning and context. Conversely, lowest-ranked systems had fundamental issues producing accurate outputs in terms of specific wording and semantic integrity.

AI-based ASR is therefore not error proof, and unnoticed residual transcription errors can pose further risks. Qualitative error analysis was crucial for understanding these (Kanal et al. 2001). Class 0 to 1 errors were generally benign, whereas class 2 errors altered the meaning of text in ways that were obvious (“the upper left K9 is measly angulated”). The primary concern stemmed from class 3 errors, which altered meaning in a way that was not obvious (mistranscription of “upper right canine” as “upper left canine”). Assessing the clinical significance of these errors revealed impactful mistakes, including incorrect tooth identification and diagnoses, altered treatment plans, and incorrect patient instructions, which were seen across systems at various levels. The identification of clinically significant errors fuels concern about automation bias, with clinicians overestimating the accuracy of an AI-generated transcript, especially after GEC has produced near-perfect general grammar and formatting (Wang et al. 2023). The challenge of detecting domain-related errors was evident during the manual error analysis; identifying mistakes such as incorrect tooth laterality when discussing extractions required vigilance, highlighting the potential for clinical harm. Beyond transcriptional accuracy, hallucinations presented a further challenge. Although not widespread, these were observed in outputs from DigitalTCO and Whisper, manifesting diversely and including the insertion of completely incoherent text or inappropriate phrases (“Thank you for watching! Subscribe to our channel!”) presumably representing training data artifacts (Metz et al. 2024). More troubling from a clinical perspective were hallucinations generating plausible but factually incorrect information, including invented discussions about tooth restorations, incorrect statements on tooth absence, alternative treatments and tooth impactions, or misinterpretation of patient instructions. These pose significant risk, because they can blend into the clinical narrative and escape detection. To date, there are no data relating to ASR systems and hallucinations in health care, but a recent study found a substantial portion of hallucinations associated with Whisper can be potentially harmful in a nonclinical context (Koenecke et al. 2024). It should also be recognized that although newer ASR and LLM systems had reduced errors and GPT4oTranscribe produced no hallucinations, the inherent stochastic nature of these systems means susceptibility remains.

Practical implementation variables also significantly influenced real-world performance. Introducing ambient clinical background noise led to varying increases in WER and DWER across systems compared with clean audio and increased class 3 clinically significant errors for each system, highlighting the importance of the acoustic environment and the necessity for noise suppression using unidirectional microphones to improve transcription fidelity. Moreover, for equitable deployment of ASR in health care, the potential for performance bias related to speaker characteristics should also be considered (Zolnoori et al. 2024). Here, performance according to accent also varied by system, even among native English speakers. The use of ASR holds much promise for streamlining clinical documentation, with ambient AI also offering the potential to record conversations between patients and clinicians, generating notes and letters (Van Veen et al. 2024). However, reliability depends on accurate transcription for multiple users, including accent type.

Responsibly integrating ASR into clinical practice requires input from clinicians and developers. Fine-tuning models on large curated datasets is key to reducing the gap between clinical terminology and general language, and incorporating comprehensive structured clinical vocabularies such as the Systematized Nomenclature of Medicine Clinical Terms (SNOMED CT) database is key to this process (NHS England 2025). Future products should feature uncertainty-aware visualizations to highlight terms with low-calibrated confidence scores for manual verification, particularly those deemed clinically significant (Loftus et al. 2022). In the clinic, the most important safeguard is maintaining a “human-in-the-loop” workflow to verify transcripts, as clinicians move from authors to editors of their notes (Altschuler et al. 2024). It is imperative to guard against automation bias, the tendency to assume accuracy of an AI-generated transcript, because it appears polished after LLM correction. Commercial companies share this responsibility and must be candid regarding limitations of the contemporary technology.

### Limitations of the Study

This study was conducted on prepared orthodontic clinical records read verbatim, which does not fully represent fluent-spoken conversational language. Orthodontic records have some terminologies that differ from general dentistry, so the results might not be generalizable to all dentistry. In addition, the systems tested will inevitably be updated, and more will enter the market. We also focused on English-language transcriptions, with the accuracy of ASR known to differ between languages (Benzeghiba et al. 2007), limiting the international generalizability of our findings. Furthermore, although the qualitative error analyses were conducted by consensus, there is inevitably some subjectivity, particularly regarding the interpretation of error impact. Future research should prioritize in vivo studies capturing real-world patient–clinician dialogue. The significant speed advantage observed in the experimental pipeline highlighted the potential efficiency gains; however, the clinical impact of these systems also needs evaluation, quantifying the time clinicians spend verifying and correcting AI-generated transcripts, reductions in documentation workload, and ultimately, whether these systems meaningfully reduce clinician workload and enhance clinician–patient interactions. Contemporary ASR systems can also improve with training and fine-tuning, although how much data is required for robust and generalizable performance is currently unknown (Latif et al. 2021). Fine-tuning Whisper in a medical context can improve an untrained model when evaluating the same dataset; however, the true generalizability of fine-tuning is poorly understood (Roushan et al. 2024). Here, we assessed only “out-of-the-box” performance and did not fine-tune either the ASR or LLM GEC step on our own data. Although this would likely reduce WER, external validity would remain uncertain without an external dataset; consequently, out-of-sample evaluation is required to ensure that improvements from fine-tuning persist beyond the original training data.

## Conclusions

This investigation revealed significant performance variability among tested ASR systems, with all capable of introducing clinically significant mistranscriptions. Clinicians using these systems should be cautious about plausible subtle substitutions or omissions of domain-specific terminology. The current status of ASR necessitates vigilance to guard against automation bias in the clinical environment, improvement in domain-specific accuracy, and potential uncertainty-aware features to ensure safe and reliable integration into clinical practice.

## Author Contributions

R. O’Kane, contributed to conception and design, data acquisition, analysis, interpretation, drafted and critically revised the manuscript; D. Stonehouse-Smith, contributed to data acquisition, interpretation, critically revised the manuscript; L.C.U. Ota, R. Patel, N. Johnson, C. Slipper, contributed to data acquisition, critically revised the manuscript; J. Seehra, contributed to data analysis, interpretation, drafted and critically revised the manuscript; S.N. Papageorgiou, contributed to data acquisition, interpretation, drafted and critically revised the manuscript; M.T. Cobourne, contributed to conception and design, data analysis and interpretation, drafted and critically revised the manuscript. All authors gave their final approval and agree to be accountable for all aspects of the work.

## Supplemental Material

sj-docx-1-jdr-10.1177_00220345251382452 – Supplemental material for Transcription Accuracy of Automatic Speech Recognition for Orthodontic Clinical Records Supplemental material, sj-docx-1-jdr-10.1177_00220345251382452 for Transcription Accuracy of Automatic Speech Recognition for Orthodontic Clinical Records by R. O’Kane, D. Stonehouse-Smith, L.C.U. Ota, R. Patel, N. Johnson, C. Slipper, J. Seehra, S.N. Papageorgiou and M.T. Cobourne in Journal of Dental Research
