# Supplementary material for: Transcription Accuracy of Automatic Speech Recognition for Orthodontic Clinical Records
Source: J Dent Res. 2025 Nov 3;105(6):737–45. doi: 10.1177/00220345251382452 (PMC13227464; doi:10.1177/00220345251382452)
Supplement: Supplementary file 1 [file sj-docx-1-jdr-10.1177_00220345251382452.docx]

**Supplementary material**

**Materials and Methods**

ASR Systems

To ensure a comprehensive assessment we selected 10 distinct Automatic Speech Recognition (ASR) systems to reflect current ASR technologies available to the international dental community. We aimed to include a representative sample of established products, including contemporary ambient Artificial Intelligence (AI) scribes, current Automatic Speech Recognition (ASR) models and an experimental hybrid ASR Large Language Model (LLM) approach. Systems only available in a beta-testing phase were excluded. The chosen systems were organised into 3 categories to evaluate different aspects of ASR performance. The first category represented directly available commercial systems (n=4). Dragon Medical One (Nuance Communications, Microsoft Corp, Burlington, USA) was selected for its specific healthcare-training along with its general-purpose counterpart, Dragon Professional Anywhere (Nuance Communications, Microsoft Corp, Burlington, USA) to evaluate the benefits of domain adaptation. We also included 2 emerging ambient-scribes marketed for use by the dental profession, Heidi Health AI Digital Scribe (Heidi Health, Sydney, Australia) and DigitalTCO (DigitalTCO, Glasgow, UK). The second category represented standalone speech-to-text Application Programming Interface (API) systems (n=5), providing direct access to foundational ASR models. These included GPT4oTranscribe (OpenAI, San Francisco, USA), Whisper OpenAI Large version 2 (OpenAI, San Francisco, USA), Amazon Medical Transcribe (Amazon Web Services, Seattle, USA), Google Medical (Google LLC, Mountain View, USA) and Speechmatics Enhanced (Speechmatics, Cambridge, UK). The third category represented an experimental ASR-LLM pipeline (n=1) used to investigate the potential of 2-stage processing through the development of an experimental pipeline, GPT4oTranscribeCorrected. This system was designed specifically to measure the impact of using a LLM for Generative Error Correction (GEC) on ASR output (see Figure 1). Together, these 10 ASR systems span the diversity of approaches available from specific dictation products to ambient scribes, foundational ASR models and LLMs whilst also covering proprietary, open-source, and hybrid architectures.

Generative Error Correction Pipeline

The transcripts produced by GPT4o Transcribe underwent a LLM GEC step (see Figure 1) with each transcript being sent to the Chat-Completions endpoint (v1/chat/completions) of the OpenAI Application Programming Interface (API) using the model gpt-4o-2024-08-06 (temperature=0, top_p=1, max_tokens=4096). The system prompt for this GEC step was optimised, a process detailed below under Prompt Optimisation.

Prompt Optimisation

For prompt optimisation, a series of prompt modification and ablation experiments were conducted on the (n=200) generated raw ASR transcripts (see Dataset Creation) from GPT4oTranscribe using the gpt-4o-2024-08-06 model at a temperature of 0 to try and allow for deterministic comparisons between prompts. Zero-shot prompting was used, which provides instructions without specific examples to avoid test set contamination. The prompt reflects established best practices in prompt-engineering (Yang et al. 2023; Ma et al. 2023; OpenAI 2024) that combine role-priming, task activation and specific constraints.

The process began by iteratively building the prompt to assess the impact of each instruction (Appendix Table 2). A simple directive (Prompt A) degraded performance relative to the baseline ASR, confirming the need for explicit instructions. Adding a role-priming (Prompt B) specifically improved accuracy on dental terminology (DWER), whilst incorporating detailed task activation (Prompt C) yielded a more substantial improvement in overall accuracy. This resulted in the final prompt (Prompt D) which added constraints on spelling (UK English), a domain cue for phonetic substitution errors and a formatting guard, achieving the lowest initial WER (3.73%) and DWER (3.45%).

To confirm the contribution of each component and test variations, we conducted a series of ablation and modifications (Prompts E-J) (Appendix Table 2). Removing instructions such as the “UK English” requirement (Prompt E), or the phonetic substitution cue (Prompt F) were seen to lower performance. These results also revealed a cluster of top-performing prompts (D, G, H, I, J) with very similar WER ranging from 3.73-3.79%. To determine if these minor differences were due to model stochasticity, we quantified the LLM baseline non-determinism by running an identical prompt 5 times, using a fixed seed (42) and temperature=0, with a within-transcript SD of 0.18% being found. Since the performance difference for the top-performing prompts were within this margin of non-determinism, we selected Prompt D as it had the lowest WER=3.73% and matched Prompt J for lowest DWER=3.45% in this specific experimental run.

After establishing the prompt, we next determined the optimal temperature parameter for accuracy and reproducibility (Appendix Table 3). To mitigate against potential bias from a single random seed, we performed five complete runs on the full (n=200) transcript dataset, each using a unique, fixed seed. This process was used to compare temperatures of 0, 0.5 and 1.0. A temperature of 0 yielded the best performance with the lowest median WER=3.78%, the LLM produced different outputs for the same transcript, with within-transcript variance being seen across 5 runs (SD 0.17%). Conversely, raising the temperature to 1.0 not only decreased accuracy (WER=4.02%) but also increased output variance more than 4-fold (SD 0.70%). Given the clinical requirement for accuracy and predictable outputs, a temperature of 0 was fixed for all subsequent analyses.

Dataset Creation

A sample of (n=200) transcripts were generated in Microsoft Word for Mac (version 16.90.2) (Microsoft Corp, Burlington, MA, USA) from anonymised clinical records to represent a library of contemporary clinical summaries relating to orthodontic diagnosis and treatment planning. The library covered 10 generic subject areas containing 20 individual transcripts within each, including: clinical and cephalometric analysis; class I, class II division 1, class II division 2 and class III malocclusion; tooth impaction; fixed appliances; syndromic conditions; orthognathic surgery; stability and retention. These transcripts yielded a combined total of 43,408 words, with minimum, maximum and mean word count per transcript of 19, 677 and 217.04 words, respectively.

Each individual transcript represented the ground truth for the experimental design, with each transcript narrated by a single individual (RO) in a closed environment using a RODE NT-USB (RØDE, Silverwater, NSW, Australia) mini-microphone connected via USB-C to MacBook Air M3 and recorded in Audacity for Mac (version 3.7.1). Audio files were recorded in [.wav] format at 16-bit depth and 44.1kHz to ensure high audio quality. The recorded audio totalled 6 hours.

Each (n=200) individual [.wav] audio file was passed through each system under investigation and used to transcribe individual text. To run audio files through the systems, for Heidi Health the [.wav] file was directly uploaded and the resulting word-for-word transcription stored; for DigitalTCO, files were first converted into the requested [.webm] service format using FFmpeg (version 0.2.0 <https://pypi.org/project/ffmpeg-python/>); for Dragon Professional Anywhere and Dragon Medical One, VB-CABLE Virtual Audio Device was used to playback the [.wav] files at 44.1kHz so quality was preserved and that the software recognised them as the input. Audio files in [.wav] were run through the APIs of Amazon Medical Transcribe (‘Primary Care, Dictation’), Whisper and GPT4o Transcribe OpenAI, Google Medical (‘medical_dictation’), Speechmatics Enhanced. All ASR systems were used with their default parameters.

Data Collation and Evaluation

The final text generated from each transcription was collated into Microsoft Excel for Mac (version 16.92) (Microsoft Corp, Burlington, MA, USA) file for subsequent evaluation.

The reference scripts (ground truth) were then compared against the generated texts using Visual Studio Code for Mac (version 1.95, Microsoft Corp, Burlington, MA, USA) with Python (<http://www.python.org/>, version 3.13). The evaluation metrics were calculated using the packages: pandas (version 2.2.2 <https://pandas.pydata.org>), torch (version 2.60+cu124 <https://pytorch.org/>) transformers (version 4.49.0, <https://github.com/huggingface/transformers>), rouge_score (version 0.1.2 <https://github.com/google-research/google-research/tree/master/rouge>), bert_score (version 0.3.13 <https://github.com/Tiiiger/bert_score>), jiwer (version 3.1.0 <https://github.com/jitsi/jiwer>) and openpyxl (version 3.1.5 <https://openpyxl.readthedocs.io>).

Pilot study investigating timing of transcript generation

A pilot study was conducted to compare the time required to generate clinical notes using the ASR-LLM pipeline (GPT4oTranscribeCorrected) versus manual typing. A subset of (n=20) transcripts was randomly selected from the main dataset of (n=200) described above using a random number generator (transcripts: 4, 13, 15, 18, 22, 39, 53, 54, 107, 111, 116, 127, 137, 146, 152, 154, 162, 165, 186, and 190 were randomly selected).

For the ASR method, the measured components included: speaking time (the audio file duration), ASR processing time using gpt-4o-transcribe, and LLM processing time for the generative error correction step with gpt-4o-2024-08-06. The total time for ASR-generated notes was the sum of the speaking time and the two processing times. For the manual method, the time required to type the same transcripts verbatim was recorded by two authors (CO and RP) using a stopwatch and Microsoft Word for Mac (version 16.90.2).

Primary outcome

Word Error Rate (WER) is a widely employed metric in ASR that quantifies transcription accuracy (see below). Although WER provides an overall measure of performance, it may insufficiently capture domain-specific nuances and does not consider the importance of individual words or the potential impact of errors (He et al. 2011). We therefore specified Domain Word Error Rate (DWER) as our primary outcome measure, because this metric extends WER by focusing on errors associated with clinical terminology, and accuracy for these terms is critical for a medical scribe.

Sample size calculation

Sample size calculation was based on pilot data testing differences across instruments and with 5 analysed transcripts. Initially, we used a one-way analysis of variance with 95% power, which indicated that only n=3 transcripts were needed. Subsequently, as we also aimed to perform pairwise comparisons and small to moderate differences between the worst performing tools, we calculated the sample needed for a t-test. For this sample size calculation, we used a mean DWER of 0.291, a standard deviation (SD) of 0.150, a difference of 1/3 SD, alpha 5% and power 90%. This indicated a sample of 170 transcripts, which was ultimately rounded up to n=200 transcripts per instrument.

Word Error Rate

WER quantifies transcription accuracy by comparing the number of substitutions, insertions and deletions to the total number of words in the reference transcript.

$$\mathrm{WER}=\frac{S+I+D}{N}\times100\%$$

Where S=number of substitutions; I=insertions; D=deletions; and N=total words in the reference text. Although WER provides an overall measure of performance, it may insufficiently capture domain-specific nuances. The normalised WER is reported.

Domain Word Error Rate

DWER extends WER by focusing on errors associated with specialised terminology, offering a more precise indicator of how effectively an ASR system handles the technical vocabulary required. In this study, we calculated DWER by focusing on tokens from a curated domain-specific clinical vocabulary derived from the Systematized Nomenclature of Medicine Clinical Terms (SNOMED CT) database, a structured clinical vocabulary for use in EHR, representing the most comprehensive and precise clinical health terminology product globally (NHS England Digital, 2025). Dental notation and additional unique terms were verified by the investigators. DWER provides a more targeted indicator of how effectively an ASR system handles the specialised vocabulary.

$$\text{DWER}=\frac{S_{d}+I_{d}+D_{d}}{N_{d}}\times100\%$$

Where *S_d_=*number of substitutions made on domain tokens; *I_d_=*number of insertions made on domain tokens; *D_d_=*number of deletions made on domain tokens; and *N_d_=*total number of domain tokens in the reference text.

Non-Domain Word Error Rate

Non-Domain WER (N-D WER) is computed by excluding all domain-specific terms. This metric evaluates the system performance on general language tokens, providing insight into accuracy outside the specialised terminology.

$$N-D \text{WER}=\frac{S_{nd}+I_{nd}+D_{nd}}{Nnd}\times100\%$$

Where *S_nd_=*number of substitutions made on non-domain tokens; *I_nd_=*number of insertions made on non-domain tokens*; D_nd_=*number of deletions made on non-domain tokens*;* and *N_nd_=*total number of non-domain tokens in the reference text.

Unnormalised Word Error Rate

The unnormalised Word Error Rate (uWER) is calculated by penalising formatting differences, such as punctuation variations and alternative transcriptions of numbers (e.g. ‘2’ versus ‘two’ or ‘II’) in addition to the errors measured by the WER. Consequently, uWER scores tend to be higher than WER scores and reflect closer adherence to the exact reference formatting.

Character Error Rate

A Character Error Rate (CER) is similar to WER but operates at a finer level of granularity by quantifying transcription errors on a per-character basis rather than per word.

$$CER=\frac{S+I+D}{N}\times100\%$$

Effect Size

To compare the performance of each tool on domain-specific versus non-domain text, we conducted Wilcoxon signed-rank tests. We then calculated effect sizes (*r*) from the test statistic using the formula:

$$r=\frac{Z}{\surd N}$$

Where *Z* is the Wilcoxon *z*‐score, and *N* is the total number of observations. A larger *r*‐value indicates greater divergence between domain‐specific and non‐domain performance (i.e. the system struggles more with the clinical terminology relative to general language); whereas a smaller *r*‐value, suggests more consistent performance across both types of content.

Lexical accuracy

The word-level precision of generated transcripts in comparison to the ground truth text was calculated using the ROUGE (Recall-Oriented Understudy for Gisting Evaluation) software package for automatic quantitative evaluation of similarity between two texts (Lin 2004). Specifically, within this package ROUGE-1 measures the overlap of unigrams (individual words) between generated text and the reference transcription, ROUGE-2 measures the overlap of bigrams (pairs of consecutive words) and ROUGE-L focuses on the Longest Common Subsequence (LCS) to determine how well the order of words in the generated transcript matches the reference text. An overall ROUGE Score was computed by aggregating ROUGE-1, ROUGE-2 and ROUGE-L.

Semantic accuracy

The retentional meaning and contextual integrity of generated text in comparison to reference text was calculated using Bidirectional Encoder Representations from Transformers (BERT) Score and Bidirectional and Auto-Regressive Transformer (BART) Score, two automatic evaluation metrics for text generation. BERT Score computes a similarity score for each token in the candidate sentence with each token in the reference sentence (Zhang et al. 2019). This gives an insight into the token-level alignment between texts; higher scores indicate stronger semantic similarity between the candidate and reference text. In contrast, BART Score employs a pre-trained BART model to estimate the likelihood of a reference given a candidate (and vice versa) (Yuan et al. 2021). This metric captures the overall fluency of the candidate text relative to the reference- higher (or less negative) BART scores suggest that the candidate text is semantically closer to the reference. These evaluation metrics, commonly used in summarisation tasks, provide complementary perspectives on semantic accuracy.

Hallucinations

A manual review of each transcript to identify any ‘hallucinations’, defined as the introduction of incorrect or misleading information by the ASR model not present in the audio. This was done manually, with the full recording listened to while reading the transcript, flagging any extraneous words or phrases that were not actually spoken. These flagged segments of text were recorded in Microsoft Word for Mac file. A hallucination rate was then calculated for each system as the proportion of transcripts containing at least one hallucination relative to the total number of transcripts.

Domain-term Missed Ratio Analysis

To evaluate performance specifically on what terms are missed, a Domain-term Missed Ratio was calculated. This analysis focused on domain tokens drawn from a custom orthodontic dictionary derived from the Systematized Nomenclature of Medicine Clinical Terms (SNOMED CT, 2024) structured clinical health terminology product for electronic health records and supplemented with additional terms verified by clinicians in the reference transcripts. Each ASR output was examined to determine whether these tokens (including recognised synonyms) were correctly transcribed. The missed ratio represents the proportion of these domain tokens that were not successfully reproduced by the ASR system, with higher values indicating poorer performance.

$$Missed Ratio=\frac{Number of domain tokens missed in ASR output}{Total number of domain tokens in reference}\times100\%$$

Example mistranscriptions were then manually reviewed to highlight specific instances of domain-token errors.

Error analysis

Transcription errors identified by the jiwer package during uWER calculation, were categorised using a modified version of Kanals error analysis (Kanal et al. 2001). Class 0 errors are formatting errors which are grammatically correct and do not change the meaning of the text; Class 1 errors are grammatically incorrect but do not change the meaning of the text; Class 2 errors produce a change in meaning, but the error is obvious and can be easily identified; Class 3 errors change the meaning in a way that is not obvious upon immediate inspection. Class 3 errors were then categorised into where they were a clinically significant error which would lead the reader to misunderstand the clinical meaning as in to potentially impact patient care (Chang et al. 2011). Two reviewers (RO and DS-S) assessed the clinical significance of each Class 3 error; any disagreements were resolved by consensus.

Normalisation

There are multiple equally valid ways to express the same concept in written text; for instance, ‘intermaxillary’ can be written as ‘inter-maxillary’ and class I can appear as ‘class 1’ or ‘class one’. These superficial variations can inflate error metrics when assessing ASR outputs. To address these challenges and ensure fair evaluation, normalisation was applied to standardise transcription outputs, focusing on reducing penalties for formatting and stylistic differences, which is common practice when evaluating word error metrics (Radford et al. 2023). Normalisation practices were adapted for the specific requirements of orthodontics. By prioritising consistent transcription of terms, errors were penalised only for genuine mistranscription.

Specifically, normalisation involved converting transcriptions into a standardised format, enabling comparable WER to be calculated across systems. Briefly, steps included case standardisation, converting all text to lowercase and expanding all contractions (informal contractions such as ‘there’s’ were expanded to their full forms, e.g. ‘there is’); numeric formatting, removing commas within numbers to standardise numerical expressions; punctuation consistency, all unnecessary punctuation was removed including periods not followed by digits and apostrophe consistency implemented, removing whitespace before apostrophes to ensure uniform handling of possessives and contractions; domain-specific unification, with terms such as ‘class 1’ or “class one” standardised to ‘class I’; hyphenation consistency, terminology with potential hyphenation such as “inter-maxillary” was unified into single-word forms, e.g. ‘intermaxillary’; and unit standardisation, with measurements standardised to abbreviations, such as ‘millimetres’ converted to ‘mm’.

Background noise simulation

To better simulate a realistic clinical environment, an augmentation step was introduced to add background noise to clean audio files. Ambient noise from a dental practice including incidental noise and background conversation, and background noise with a radio playing were sourced from freesound.org and merged using GarageBand (version 10.4.12, Apple Inc, Cupertino, CA, USA). Using Python (<http://www.python.org/>, version 3.11) with the librosa (version 0.11.0 <https://pypi.org/project/librosa/>) and soundfile (version 0.13.1, <https://pypi.org/project/soundfile/>) libraries. Both the clean audio and noise samples were loaded with a matching sample rate; if the noise sample was shorter than the clean audio, it was looped to match the full duration of the recording. The noise signal was then scaled so that, when combined with the clean signal, the resulting audio achieved a signal-to-noise ratio (SNR) of 15 dB, recognised as the optimal level for clear speech intelligibility (Bradley et al. 1999). The augmented noisy audio files were saved in a separate directory and subsequently processed through the same ASR systems as the original clean recordings to enable direct performance comparisons.

Accent Check

To assess the potential influence of speaker accent on ASR performance, the subset of (n=20) transcripts used in the pilot study investigating timing of transcript generation were re-recorded by speakers with different accents.

In addition to the primary narrator (RO, a male native English speaker with a Northern Irish accent), two additional native English speakers were recruited: speaker CO (female, British Nigerian heritage) and speaker RP (female, British Indian heritage) recorded under the same conditions. The resulting 40 audio files (20 transcripts x2 additional speakers). Subsequently, background noise was added to these 40 clean audio files using the same procedure described previously, creating an additional 40 audio files for analysis. Both the clean (n=40) and background noise (n=40) audio files generated by speakers CO and RP were then processed using all n=10 systems under investigation. The transcription process for each followed the same protocol as the main dataset, and using the same evaluation metrics. The statistical analysis was performed by comparing metrics obtained for speakers CO and RP against those for the primary narrator (RO) on the identical subset of 20 transcripts for both clean and noisy conditions.

**Statistical analysis**

After testing for normality with the Shapiro-Wilk test, descriptive statistics were calculated included medians with Interquartile Ranges (IQR) or absolute/relative frequencies. Differences between DWER and N-D WER for each tool were investigated using the Wilcoxon-signed test, followed by effect size estimation. Differences across tools were assessed with quantile regression with robust and clustered standard errors to account for the same transcripts being used, and were expressed as unstandardized regression coefficients with their 95% Confidence Intervals (CI). After overall differences across all tools with Wald-type tests, pairwise comparisons to the best-performing tool were done with Bonferroni correction. All analyses were run in Stata SE 18.5 (StataCorp, College Station, TX, USA) with alpha set at 5% and an open-source dataset: [https://doi.org/10.5281/zenodo.15470163](https://eur03.safelinks.protection.outlook.com/?url=https%3A%2F%2Fdoi.org%2F10.5281%2Fzenodo.15470163&data=05%7C02%7Cmartyn.cobourne%40kcl.ac.uk%7C2a85cdef2ea3438332be08dd97790efe%7C8370cf1416f34c16b83c724071654356%7C0%7C0%7C638833268402900630%7CUnknown%7CTWFpbGZsb3d8eyJFbXB0eU1hcGkiOnRydWUsIlYiOiIwLjAuMDAwMCIsIlAiOiJXaW4zMiIsIkFOIjoiTWFpbCIsIldUIjoyfQ%3D%3D%7C0%7C%7C%7C&sdata=Deg1C9Zs%2BoZXWzCsJGTlFvdDo4uqAtTLoIOVV7ZT1hA%3D&reserved=0).

**References**

Bradley JS, Reich RD, Norcross SG. 1999. On the combined effects of signal-to-noise ratio and room acoustics on speech intelligibility. J Acoust Soc Am 106(4):1820-1828.

Chang CA, Strahan R, Jolley D. 2011. Non-clinical errors using voice recognition dictation software for radiology reports: A retrospective audit. J Digit Imaging. 24(4):724-728.

He X, Deng L, Acero A. 2011. Why word error rate is not a good metric for speech recognizer training for the speech translation task? 2011 IEEE International Conference on Acoustics, Speech and Signal Processing (ICASSP).

Kanal KM, Hangiandreou NJ, Sykes AM, Eklund HE, Araoz PA, Leon JA, Erickson BJ. 2001. Initial evaluation of a continuous speech recognition program for radiology. J Digit Imaging. 14(1):30-37.

Lin C-Y. 2004. ROUGE: A package for automatic evaluation of summaries. In *Text Summarization Branches Out*, pp 74–81, Barcelona, Spain. Association for Computational Linguistics.

Ma R, Qian M, Manakul P, Gales M, Knill K. 2023. Can generative large language models perform ASR error correction? arXiv:2307.04172.

NHS England Digital. 2025. SNOMED-CT NHS England Clinical Terminology Server. Available from: https://digital.nhs.uk/services/terminology-and-classifications/snomed-ct (Accessed 7^th^ August 2025).

OpenAI. 2024. Text generation and prompting guide—Chat completions endpoint. <https://platform.openai.com/docs/guides/text?api-mode=chat>. (accessed 27 Jun 2025).

Radford A, Kim JW, Xu T, Brockman G, McLeavey C, Sutskever I. 2023. Robust speech recognition via large-scale weak supervision. [arXiv:2212.04356](https://arxiv.org/abs/2212.04356)

Yang CH, Gu Y, Liu YC, Ghosh S, Bulyko I, Stolcke A. 2023. Generative speech recognition error correction with large language models and task-activating prompting. arXiv:2309.15649.

Yuan W, Neubig G, Liu P. 2021. Bartscore: Evaluating generated text as text generation. Adv Neural Inf Process. 34: 27263-27277.

Zhang T, Kishore V, Wu F, Weinberger KQ, Artzi Y. 2019. Bertscore: Evaluating text generation with bert. arXiv: 190409675.

**Appendix Table 1	List of acronyms used in the manuscript**

**AI,** Artificial Intelligence

**API,** Application Programming Interface

**ASR,** Automatic Speech Recognition

**BART score,** Bidirectional and Auto-Regressive Transformer score

**BERT score,** Bidirectional Encoder Representations from Transformers score

**CER,** Character Error Rate

**DWER,** Domain Word Error Rate

**EHR,** Electronic Health Records

**GEC,** Generative Error Correction

**LLM,** Large Language Model

**LCS,** Longest Common Sequence

**N-D WER,** Non-Domain Word Error Rate

**NLP**, Natural Language Processing

**ROUGE score,** Recall-Oriented Understudy for Gisting Evaluation

**uWER,** unnormalised Word Error Rate

**WER,** Word Error Rate

**Appendix Table 2**	Pilot data on speaking, processing, and typing times for a pilot study of 20 transcripts

| **Analysis** | **Outcome** | **Metric** | **Value** |
| --- | --- | --- | --- |
| Per transcript | Speaking time (sec) | Mean (SD) | 133.04 (65.55) |
|  | ASR processing time (sec) | Median (IQR) | 9.08 (7.66,12.12) |
|  | LLM processing time (sec) | Median (IQR) | 3.92 (2.85, 4.53) |
|  | Total processing time (sec) | Median (IQR) | 12.90 (10.26, 16.30) |
|  | Total time for ASR notes (sec) | Mean (SD) | 147.73 (71.21) |
|  | Total typing time (sec) | Mean (SD) | 364.77 (182.91) |
|  | Difference with ASR (sec) | Difference in means  (95% CI) | -217.04 (-269.57, -164.51)* |
|  | Difference with ASR (%) | Median (IQR) | -59.41% (-60.72%, -58.95%) |
|  |  |  |  |
| Per word | Total time for ASR notes (sec) | Mean (SD) | 0.47 (0.03) |
|  | Typing time (sec) | Mean (SD) | 1.16 (0.11) |
|  | Difference with ASR (sec) | Difference in means  (95% CI) | -0.69 (-0.73, -0.65)* |
|  | Difference with ASR (%) | Median (IQR) | -59.41% (-60.72%, -58.95%) |

* P-value < 0.001 for paired t-test

ASR, automatic speech recognition; CI, confidence interval; IQR, interquartile range; LLM, large language model; SD, standard deviation; sec, seconds

Data is given as the average from 2 different speakers (CO, RP). Mean (SD) transcript length: 314.1 (148.4) words (range 89 to 677)

**Appendix Table 3	Prompt-construction and validation**

| **Prompt** | **Description** | **Key instruction (s)** | **WER (%)** | **DWER (%)** | **N-DWER (%)** |
| --- | --- | --- | --- | --- | --- |
| Base | GPT4o Transcribe (Raw ASR) | - | 5.13 | 7.69 | 5.32 |
| A | Baseline GEC: minimal directive to correct the text. | Correct the following text given from an ASR. | 5.84 | 4.96 | 6.10 |
| B | Adds a role for the specific domain. | Adds: “You are a helpful assistant for transcribing dental notes.” | 4.94 | 4.57 | 5.37 |
| C | Adds detailed “task activation” to guide correction behaviour, replacing the simple directive. | Replaces simple directive with: “Your task is to carefully read the transcribed text and correct any spelling discrepancies..” | 4.72 | 4.82 | 5.08 |
| **D** | **Full prompt (final version): combines role, task activation, domain cue and specific constraints.** | **Full prompt includes all constraints: UK English, punctuation/hallucination guards, phonetic substitution cue and return format guard.** | **3.73** | **3.45** | 3.90 |
| E | Ablation from J: Removes the ‘UK English’ constraint. | Removes: “All spelling must be in UK English”. | 4.60 | 3.92 | 4.66 |
| F | Ablation from J: Removes the cue to handle phonetic substitutions. | Removes: “You will see phonetic substitution errors; these errors must be corrected to the correct dental term...” | 3.91 | 3.67 | 4.17 |
| G | Ablation from J: Removes the return format guard | Removes “Return only the complete, corrected transcript text”. | 3.76 | 3.67 | **3.88** |
| H | Ablation from J: Removes initial role line | Removes: “You are a helpful assistant for transcribing dental notes.” | 3.75 | 3.64 | 4.05 |
| I | Modification of J: Replaces the domain cue ‘dental term’ with ‘SNOMED-CT terms’. | Replaces: 'dental term' with 'SNOMED-CT terms' in the phonetic substitution instruction. | 3.75 | 3.70 | 3.90 |
| J | Modification of J: Replaces 'dental term' with 'orthodontic/dental terminology'. | Replaces: 'dental term' with 'orthodontic/dental terminology' in the phonetic substitution instruction. | 3.79 | **3.45** | 4.02 |

GEC, generative error correction; WER, word error rate; DWER, domain word error rate; N-DWER, non-domain word error rate

**Appendix Table 4	Effect of model temperature on accuracy and output reproducibility**

| Temperature | Median WER (%) | Median DWER (%) | *Within-transcript SD WER (%) | *Within-transcript SD DWER (%) |
| --- | --- | --- | --- | --- |
| 0 | 3.78 | 3.45 | 0.17 | 0.28 |
| 0.5 | 3.82 | 3.45 | 0.44 | 0.69 |
| 1.0 | 4.02 | 3.64 | 0.70 | 1.44 |

Accuracy metrics (median word error rate (WER), median domain word error rate (DWER): median values are calculated from the 1,000 resulting transcriptions (200 transcripts x 5 runs) for each temperature setting

Reproducibility (Within-transcript SD): The standard deviation (SD) was calculated on error rates from five runs for each transcript and then averaged across the 200 transcripts per run. This quantifies output variability; with lower values indicating higher reproducibility

**Appendix Table 5**	**Pairwise comparison of ASR systems for word and character error outcomes**

| **ASR system** | **WER** | | **DWER** | | **N-D WER** | | **uWER** | | **CER** | |
| --- | --- | --- | --- | --- | --- | --- | --- | --- | --- | --- |
|  | **b (95% CI)** | **P-value*** | **b (95% CI)** | **P-value*** | **b (95% CI)** | **P*-value** | **b (95% CI)** | **P*-value** | **b (95% CI)** | **P*-value** |
| GPT4o Transcribe Corrected | Reference |  | Reference |  | Reference |  | Reference |  | Reference |  |
| Amazon Medical Transcribe (API) | 8.60  (7.76, 9.43) | <0.001* | 20.04  (17.49, 22.59) | <0.001* | 10.21  (9.06, 11.37) | <0.001* | 14.27  (13.11, 15.42) | <0.001* | 4.25  (3.73, 4.76) | <0.001* |
| DigitalTCO | 2.72  (2.05, 3.39) | <0.001* | 9.01  (6.46, 11.56) | <0.001* | 3.15  (2.45, 3.86) | <0.001* | 3.79  (2.89, 4.69) | <0.001* | 1.35  (1.06, 1.65) | <0.001* |
| Dragon Medical One | 20.46  (18.52, 22.40) | <0.001* | 26.51  (24.24, 28.78) | <0.001* | 22.40  (20.51, 24.28) | <0.001* | 28.11  (27.04, 29.18) | <0.001* | 10.56  (9.98, 11.13) | <0.001* |
| Dragon Professional Anywhere | 30.25  (28.98, 31.52) | <0.001* | 45.16  (42.61, 47.71) | <0.001* | 33.39  (31.70, 35.09) | <0.001* | 36.00  (34.88, 37.11) | <0.001* | 15.83  (14.94, 16.71) | <0.001* |
| Google Medical (API) | 7.26  (6.41, 8.12) | <0.001* | 19.92  (17.01, 22.82) | <0.001* | 7.33  (6.43, 8.23) | <0.001* | 8.61  (7.61, 9.61) | <0.001* | 3.92  (3.48, 4.36) | <0.001* |
| GPT4o Transcribe | 1.39  (0.96,1.83) | <0.001* | 4.20  (2.70, 5.71) | <0.001* | 1.43  (0.95, 1.91) | <0.001* | 1.25  (0.66, 1.84) | <0.001* | 0.40  (0.25, 0.56) | <0.001* |
| Heidi Health | 1.72  (1.23, 2.21) | <0.001* | 2.76  (1.14, 4.39) | 0.009* | 1.58  (0.99, 2.18) | <0.001* | 1.63  (0.89, 2.36) | <0.001* | 0.84  (0.59, 1.08) | <0.001* |
| Speechmatics Enhanced (API) | 5.36  (4.50, 6.21) | <0.001* | 12.07  (9.81, 14.32) | <0.001* | 6.10  (5.22, 6.99) | <0.001* | 8.95  (7.74, 10.15) | <0.001* | 2.20  (1.89, 2.52) | <0.001* |
| Whisper OpenAI Large version 2 (API) | 7.28  (5. 42, 9.14) | <0.001* | 12.64  (10.70, 14.58) | <0.001* | 7.47  (5.95, 8.98) | <0.001* | 7.94  (5.46, 10.43) | <0.001* | 4.03  (3.19, 4.86) | <0.001* |

b, regression coefficient; CER, character error rate; CI, confidence interval; DWER, domain word error rate; N-DWER, non-domain word error rate; uWER, unnormalised word error rate; WER, word error rate

* Bonferroni correction applied

**Appendix Table 6	Comparison of different ROUGE metrics by ASR system**

| **ASR system** | **ROUGE-1**  Median  (IQR) | **ROUGE-2**  Median  (IQR) | **ROUGE-L**  Median  (IQR) |
| --- | --- | --- | --- |
| Amazon Medical Transcribe *(API)* | 0.904  (0.872, 0.933) | 0.821  (0.768, 0.868) | 0.901  (0.869, 0.927) |
| DigitalTCO | 0.953  (0.931, 0.968) | 0.904  (0.867, 0.937) | 0.951  (0.930, 0.967) |
| Dragon Medical One | 0.835  (0.800, 0.866) | 0.676  (0.623, 0.726) | 0.815  (0.778, 0.852) |
| Dragon Professional Anywhere | 0.765  (0.720, 0.807) | 0.571  (0.505, 0.635) | 0.735  (0.680, 0.780) |
| Google Medical *(API)* | 0.916  (0.888, 0.937) | 0.837  (0.788, 0.878) | 0.912  (0.881, 0.935) |
| GPT4o Transcribe | 0.960  (0.942, 0.976) | 0.923  (0.885, 0.952) | 0.960  (0.940, 0.976) |
| GPT4o Transcribe Corrected | 0.970  (0.955, 0.982) | 0.940  (0.909, 0.963) | 0.969  (0.953, 0.982) |
| Heidi Health | 0.963  (0.947, 0.976) | 0.921  (0.891, 0.947) | 0.960  (0.944, 0.974) |
| Speechmatics Enhanced *(API)* | 0.926  (0.905, 0.951) | 0.858  (0.822, 0.898) | 0.924  (0.902, 0.946) |
| Whisper OpenAI Large version 2 *(API)* | 0.926  (0.881, 0.950) | 0.869  (0.814, 0.912) | 0.923  (0.870, 0.949) |
| P-value | <0.001* | <0.001* | <0.001* |

* Wald-type test for overall differences from quantile regression

IQR, interquartile range; ROUGE, recall-oriented understudy for gisting evaluation

**Appendix Table 7**	**Post hoc pairwise comparison of ASR systems for lexical and semantic outcomes**

| **ASR system** | **ROUGE** | | **ROUGE-1** | | **ROUGE-2** | | **ROUGE-L** | | **BART** | | **BERT** | | |
| --- | --- | --- | --- | --- | --- | --- | --- | --- | --- | --- | --- | --- | --- |
|  | **b (95% CI)** | **P-value*** | **b (95% CI)** | **P-value*** | **b (95% CI)** | **P-value*** | **b (95% CI)** | **P-value*** | **b (95% CI)** | **P-value*** | **b (95% CI)** | **P-value*** |  |
| GPT4o Transcribe Corrected | Reference |  | Reference |  | Reference |  | Reference |  | Reference |  | Reference |  |  |
| Amazon Medical Transcribe (API) | -0.08  (-0.09, -0.08) | <0.001* | -0.07  (-0.07, -0.06) | <0.001* | -0.12  (-0.13, -0.11) | <0.001* | -0.07  (-0.07, -0.06) | <0.001* | -0.81  (-0.87, -0.76) | <0.001* | -0.08  (-0.09, -0.07) | <0.001* |  |
| DigitalTCO | -0.02  (-0.03, -0.02) | <0.001* | -0.02  (-0.02, -0.01) | <0.001* | -0.04  (-0.04, -0.03) | <0.001* | -0.02  (-0.02, -0.01) | <0.001* | -0.30  (-0.36, -0.24) | <0.001* | -0.03  (-0.03, -0.02) | <0.001* |  |
| Dragon Medical One | -0.19  (-0.20, -0.18) | <0.001* | -0.14  (-0.14, -0.13) | <0.001* | -0.26  (-0.28, -0.25) | <0.001* | -0.16  (-0.16, -0.15) | <0.001* | -1.72  (-1.87, -1.56) | <0.001* | -0.19  (-0.19, -0.18) | <0.001* |  |
| Dragon Professional Anywhere | -0.27  (-0.28, 09,26) | <0.001* | -0.20  (-0.21, -0.20) | <0.001* | -0.37  (-0.38, -0.35) | <0.001* | -0.23  (-0.25, -0.22) | <0.001* | -2.88  (-3.04, -2.72) | <0.001* | -0.24  (-0.25, -0.23) | <0.001* |  |
| Google Medical (API) | -0.07  (-0.08, -0.07) | <0.001* | -0.05  (-0.06, -0.05) | <0.001* | -0.10  (-0.12, -0.09) | <0.001* | -0.06  (-.06, -0.05) | <0.001* | -0.65  (-0.71, -0.60) | <0.001* | -0.06  (-0.06, -0.05) | <0.001* |  |
| GPT4o Transcribe | -0.01  (-0.02, -0.01) | <0.001* | -0.01  (-0.01, -0.01) | <0.001* | -0.02  (-0.03, -0.01) | <0.001* | -0.01  (-0.01, 0) | <0.001* | -0.12  (-0.15, -0.09) | <0.001* | -0.01  (-0.01, 0) | 0.001* |  |
| Heidi Health | -0.01  (-0.02, -0.01) | <0.001* | -0.01  (-0.01, 0)) | 0.008* | -0.02  (-0.03, -0.01) | <0.001* | -0.01  (-0.01, 0) | <0.001* | -0.14  (-0.17, -0.10) | <0.001* | -0.01  (-0.02, -0.01) | <0.001* |  |
| Speechmatics Enhanced (API) | -0.06  (-0.06, -0.05) | <0.001* | -0.04  (-0.05, -0.04) | <0.001* | -0.08  (-0.09, -0.07) | <0.001* | -0.05  (-0.05, -0.04) | <0.001* | -0.48  (-0.54, -0.42) | <0.001* | -0.04  (-0.05, -0.04) | <0.001* |  |
| Whisper OpenAI Large version 2 (API) | -0.06 (-0.07, -0.04) | <0.001* | -0.04  (-0.05, -0.03) | <0.001* | -0.07  (-0.09, -0.06) | <0.001* | -0.05  (-0.06, -0.04) | <0.001* | -0.48  (-0.54, -0.43) | <0.001* | -0.04  (-0.05, -0.03) | <0.001* |  |

b, regression coefficient; CER, character error rate; CI, confidence interval; DWER, domain word error rate; N-D WER, non-domain word error rate; uWER, unnormalised word error rate; WER, word error rate

* Bonferroni correction applied.

**Appendix Table 8	Examples of hallucinations in ASR system-generated text**

| **ASR system** | **Transcript number** | **Reference text (ground truth)** | **Generated text** |
| --- | --- | --- | --- |
| **Whisper OpenAI Large version 2** | 15 | The class 2 skeletal pattern, 9mm overjet, and unilateral crossbite further complicate management.  The proposed treatment plan involves preserving the carious first permanent molars temporarily to maintain space during the initial phase of treatment. | The class three skeletal pattern, 9 mm overjet and unilateral crossbed further complicate the problem**.** **With the LL7 and UL6 having a worse prognosis than the LR6. And severe crowding in the upper and lower arches. The upper premolars and canines are impacted with the UL5 and UR5 positioned palatally and the UL3 buccally crowded.** further complicate management**.** The proposed treatment plan involves preserving the carious first permanent molars temporarily to maintain space during the initial phase of treatment |
| **Whisper OpenAI Large version 2** | 20 | Extra-oral examination showed a convex profile with a moderate class 2 skeletal base. The Frankfort-mandibular planes angle was average, and the anterior lower face height was reduced. No significant transverse facial asymmetry was observed. | Extraoral examination showed a convex profile with a moderate Class II skeletal base. The Frankfurt mandibular plane's angle was average and the anterior lower face height was reduced. **The lower left lateral incisor and canine were restored to normal.** No significant transverse facial asymmetry was observed. |
| **DigitalTCO** | 19 | Arrangements will be made for extractions and subsequent reviews to ensure proper dental development. *End of dictation* | Arrangements will be made for extractions and subsequent reviews to ensure proper dental development. **For more information visit www.fbe.ca** |
| **Whisper OpenAI Large version 2** | 27 | The patient was instructed to wear the facemask for a minimum of 16 hours daily. | The patient was instructed to wear the face mask for a minimum of 10 minutes. **The patient was instructed to wear the face mask for a minimum of 10 minutes.** |
| **Whisper OpenAI Large version 2** | 122 | The discussion focused on the need for an alveolar bone graft to address the maxillary alveolar defect associated with the cleft. The procedure is planned for around the age of 10 to11, shortly before the eruption of the permanent canine teeth. This timing is critical to ensure the graft provides sufficient support for the canines to erupt through the cleft site and enables effective dental and orthodontic management. | The discussion focused on the need for an alveolar bone graft to address the maxillary alveolar defect associated with the cleft. The procedure is planned for around the age of 10-11 shortly before the eruption of the permanent canine teeth. This timing **is important because the alveolar bone graft is not a normal bone graft.** The procedure is critical to ensure the graft provides sufficient support for the canines to erupt through the cleft site and enables effective dental and orthodontic management. |
| **Whisper OpenAI Large version 2** | 160 | At debonding, a bonded retainer was placed in the lower arch to maintain alignment, and the patient was provided with removable vacuum-formed retainers to wear at night. Emily completed her active treatment in 22 months, achieving a well-aligned and functional occlusion with a stable result. | At debonding, a bonded retainer was placed in the lower arch to maintain alignment. Vacuum formed retainers were used to wear at night. Emily completed her active treatment in 22 months achieving a well lined and functional occlusion with a stable result. **Thank you for watching. Please subscribe to our channel for more videos. Thank you for watching.** |
| **Whisper OpenAI Large version 2** | 197 | Where brackets are not repositioned, round stainless steel archwires (0.018-inch) or TMA archwires may be considered as they are formable and have sufficient flexibility and range to make minor adjustments. Where brackets have been repositioned either nickel titanium, TMA or light stainless steel archwires might be considered. | Where brackets are not repositioned around stainless steel archwires, **0.016-inch nickel titanium**, 0.018 inch or TMA archwires may be considered as they are formable and have sufficient flexibility and range to make minor adjustments. Where brackets have been repositioned, either nickel titanium, TMA, or light stainless steel archwires might be considered. |

This table presents instances where ASR systems produced hallucinations. The table includes the ASR system responsible for the hallucination, transcript number, reference text (representing the ground truth) and generated text (form the ASR system). Hallucinated text within the generated output is highlighted in bold

**Appendix Table 9	Examples of Kanal error categories**

| Spoken phrase | Class 0 Error | Class 1 Error | Class 2 Error | Clinically significant Class 3 Error | Clinically not significant Class 3 Error |
| --- | --- | --- | --- | --- | --- |
|  | Formatting errors | Grammatical errors | Change in meaning which is obvious | Alters the meaning in a manner which is not obvious and could potentially impact patient care. | Alters the meaning in a way that is not obvious and does not potentially impact patient care. |
| ‘The upper left canine is mesially angulated’. | ‘The upper left canine mesially**-**angulated’. | ‘The upper left canine is mesially **angulation’.** | ‘The upper left **K9** is **measly** angulated’ | ‘The upper **right** canine is mesially angulated’. | ‘The upper left canine is **mildly** angulated’. |
| ‘In this instance, the displacement between RCP and ICP was greater than 2mm, assigning an IOTN of 4c’. | ‘In this instance, the displacement between **rcp** and **icp** was greater than 2 millimetre assigning an **iotn** of 4C’. | ‘In this **instances**, the **displacements** between RCP and ICP were greater than 2mm, assigning an IOTN of 4.c’. | ‘In this instance, the displacement between RCP and ICP was greater than 2mm, assigning an IOTN of **four sea**’. | ‘In this instance, the displacement between RCP and ICP was **not** greater than 2mm, assigning an IOTN of **2c**’. | ‘In this instance, the displacement between RCP and ICP was greater than 2mm, assigning an IOTN of 4**d**’. |

Class 0 Error: formatting errors which are grammatically correct and do not change the meaning of the text

Class 1 Error: errors are grammatically incorrect but do not change the meaning of the text

Class 2 Error: errors produce a change in meaning, but the error is obvious and can be easily identified

Clinically significant Class 3 error: changes the meaning in a way that is not obvious upon immediate inspection, which potentially impacts patient care

Clinically not significant Class 3 Error: changes the meaning in a way that is not obvious upon immediate inspection, which does not potentially impact patient care

**Appendix Table 10	Summary of class 3 clinically significant errors across tools (clean versus background noise)**

| **Tool** | **Total errors**  (n) | | **Number of Class 3 clinically significant errors**  (n) | | **Proportion of errors that are Class 3 clinically significant**  (%) | | **Proportion of transcripts with at least 1 clinically significant error (%)** | |
| --- | --- | --- | --- | --- | --- | --- | --- | --- |
|  | Clean | Background Noise | Clean | Background Noise | Clean | Background Noise | Clean | Background Noise |
| GPT4o Transcribe Corrected | 1,949 | 1,995 | 4 | 10 | 0.21 | 0.50 | 2 | 3.5 |
| GPT4o Transcribe | 2,497 | 2,474 | 8 | 12 | 0.32 | 0.49 | 3.5 | 5 |
| Heidi Health | 2,755 | 3,454 | 28 | 55 | 1.02 | 1.58 | 10.5 | 22.50 |
| DigitalTCO | 3,150 | 3,716 | 79 | 78 | 2.51 | 2.09 | 23 | 23.50 |
| Speechmatics Enhanced | 4,424 | 4,866 | 122 | 126 | 2.76 | 2.59 | 27 | 31.5 |
| Google Medical | 5,480 | 6,002 | 134 | 159 | 2.45 | 2.65 | 39.5 | 44.5 |
| Amazon Medical Transcribe | 5,897 | 6,573 | 119 | 129 | 2.02 | 1.96 | 31.5 | 38 |
| Whisper OpenAI Large version 2 | 7,257 | 7,786 | 192 | 199 | 2.65 | 2.56 | 40.5 | 45.50 |
| Dragon Medical One | 10,728 | 13,664 | 353 | 567 | 3.29 | 4.15 | 53 | 66 |
| Dragon Professional Anywhere | 14,480 | 16,679 | 340 | 420 | 2.35 | 2.52 | 47 | 51.50 |

Total Errors: The sum of Class 0-3 errors for each tool and condition

Proportion of errors that are Class 3 clinically significant: among all errors, the percentage that are Class 3 and deemed clinically significant = [number of clinically significant Class 3 errors ÷ total errors] x 100.

Proportion of transcripts with at least 1 Class 3 clinically significant error: indicates what proportion of transcripts had at least one clinically significant error out of (n=200) transcripts per ASR system/condition

**Appendix Table 11**	**Examples of Class 3 clinically significant errors across domain categories**

| Domain Category | Example | | % of Class 3 clinically significant errors |
| --- | --- | --- | --- |
|  | **Reference Text** | **Generated Text by ASR** |  |
| Tooth Identification and Numbering | *‘The LR6 or LR4 will also be extracted based on caries severity or hypoplasia’.* | *‘The* ***LL6*** *or* ***LL4*** *will also be extracted based on caries severity or hypoplasia’.-* DigitalTCO, Background Noise, Transcript #15 | 12.32 |
| Malocclusion and Skeletal Assessment | *‘The skeletal relationship is consistent with a moderate skeletal Class 2 pattern’.* | *‘The skeletal relationship is consistent with a moderate skeletal* ***class III*** *pattern’-* Heidi Health, Background Noise, Transcript #165 | 17.02 |
| Dental anomalies and development | ***‘****Patient is in the mixed dentition stage’.* | *‘Patient is* ***an excellent pleasant state’.****- Dragon Medical One, Clean, Transcript #19* | 7.99 |
| Clinical Examination and findings | ***‘****Lip competence is good’.* | *‘Patient has* ***incomplete lip competence’.****-* Dragon Professional Anywhere, Clean, Transcript #4 | 39.80 |
| Treatment and Procedural Recommendations | **‘**Correct the incisor relationship’. | *‘Correct the* ***symptoms’.****-* Whisper OpenAI Large version 2 (API), Clean, Transcript #13 | 12.03 |
| Negation and affirmation | ‘With reasonable root length, no restorations, no mobility, and no symptoms’. | *‘With reasonable root length,* ***new*** *restorations,* ***new*** *mobility, and* ***new*** *symptoms’.*  Speechmatics, Background Noise, Transcript #13 | 3.07 |
| Patient history and background Information | *‘She reported no significant issues with eating. Her medical history included well-controlled eczema, a single episode of pityriasis rosea, a family history of sickle cell disease, and carrier status for haemoglobin C’.* | *‘She reported no significant issues with eating. Her medical history included well-controlled eczema, a single episode of pityriasis, a family history of sickle cell disease, and* ***caries on deciduous teeth’.****-* Heidi Health, Background Noise, Transcript #114 | 2.63 |
| Medication, devices and appliances | ***‘****Protraction headgear”* | ***‘Retraction*** *headgear”*- Heidi Health, Clean, Transcript 28 | 4.54 |
| Patient communication and instructions | *‘The patient was instructed to wear the facemask for a minimum of 16 hours daily’.* | *‘The patient was instructed to wear the face mask for a minimum of* ***10 minutes’.****-* Whisper OpenAI Large version 2 (API), Clean, Transcript #27 | 0.6 |

Domain Category: classification of the error’s clinical context

Reference Text: exact excerpt of the spoken content

Generated Text: ASR system-generated transcript containing the error; the errors are shown in bold

% of Class 3 clinically significant errors: percentage of all Class 3 clinically significant errors attributable to this domain

**Appendix Table 12**	**Comparative performance of the various ASR systems according to the presence of background noise**

|  |  | **Comparison within instrument – background noise versus clean** | |
| --- | --- | --- | --- |
| **Outcome** | **ASR system** | **b (95% CI)** | **P-value*** |
| WER | GPT4oTranscribe Corrected | 0 (0, 0.01) | 1.00* |
|  | Amazon Medical Transcribe | 0.02 (0.01, 0.03) | <0.001* |
|  | DigitalTCO | 0.01 (0.01, 0.02) | 0.002* |
|  | Dragon Medical One | 0.09 (0.06, 0.11) | <0.001* |
|  | Dragon Professional Anywhere | 0.07 (0.04, 0.09) | <0.001* |
|  | Google Medical | 0.03 (0.02, 0.03) | <0.001* |
|  | GPT4oTranscribe | 0 (0, 0) | 1.00* |
|  | Heidi Health | 0.01 (0.01, 0.02) | <0.001* |
|  | Speechmatics Enhanced | 0.01 (0, 0.01) | 0.18* |
|  | Whisper OpenAI Large version 2 | 0.02 (0, 0.04) | 0.12* |
|  |  |  |  |
| DWER | GPT4oTranscribe Corrected | 0 (-0.01, 0.02) | 1.00* |
|  | Amazon Medical Transcribe | 0.01 (0, 0.03) | 0.90* |
|  | DigitalTCO | 0.03 (0.01, 0.04) | 0.03* |
|  | Dragon Medical One | 0.10 (0.07, 0.13) | <0.001* |
|  | Dragon Professional Anywhere | 0.05 (0.02, 0.08) | 0.003* |
|  | Google Medical | 0.07 (0.04, 0.09) | <0.001* |
|  | GPT4o Transcribe | 0 (-0.02, 0.02) | 1.00* |
|  | Heidi Health | 0.01 (0, 0.03) | 0.98* |
|  | Speechmatics Enhanced | 0.01 (0, 0.03) | 1.00* |
|  | Whisper OpenAI Large version 2 | 0.05 (0.03, 0.07) | <0.001* |

b, unstandardized regression coefficient; CI, confidence interval; DWER, domain word error rate; WER, word error rate.

*P-value corrected for multiple testing on the level of the outcome

In the presence of background noise, GPT4oTranscribeCorrected, GPT4oTranscribe and Speechmatics Enhanced showed no significant change in WER (≤0.01%, P≥0.18) and non-significant change in DWER (≤0.01%, P≥0.98). Heidi Health exhibited only a 0.01 WER increase (P<0.001). In contrast, Dragon Medical One and Dragon Professional Anywhere were most affected, with WER increases of 0.09% and 0.07% and DWER of 0.10% and 0.05%, respectively. Google Medical also exhibited a notable DWER increment (0.07%) despite only a moderate WER rise (0.03%). Whisper maintained WER (0.02%, P=0.12) but still displayed a significant DWER increase (0.05%, P<0.001).

**Appendix Table 13	WER of the ASR systems according to the speaker accent and stratified by background noise. Non-significant interaction terms of system x accent for clean (P=0.19) and background noise (P=0.31)**

|  |  | **Clean** | |  | **Background noise** | |
| --- | --- | --- | --- | --- | --- | --- |
|  |  | **b (95% CI)** | **P-value*** |  | **b (95% CI)** | **P-value*** |
| ASR system | GPT4o Transcribe Corrected | Reference | <0.001 |  | Reference | <0.001 |
|  | Amazon Medical Transcribe | 0.12 (0.10, 0.14) |  |  | 0.13 (0.11, 0.15) |  |
|  | DigitalTCO | 0.04 (0.02, 0.05) |  |  | 0.04 (0.02, 0.05) |  |
|  | Dragon Medical One | 0.18 (0.14, 0.22) |  |  | 0.47 (0.42, 0.51) |  |
|  | Dragon Professional Anywhere | 0.27 (0.25, 0.30) |  |  | 0.48 (0.43, 0.53) |  |
|  | Google Medical | 0.08 (0.06, 0.10) |  |  | 0.10 (0.08, 0.11) |  |
|  | GPT4o Transcribe | 0.02 (0.01, 0.04) |  |  | 0.02 (0, 0.03) |  |
|  | Heidi Health | 0.01 (-0.01, 0.02) |  |  | 0.02 (0.01, 0.04) |  |
|  | Speechmatics Enhanced | 0.05 (0.03, 0.07) |  |  | 0.05 (0.03, 0.07) |  |
|  | Whisper OpenAI Large version 2 | 0.06 (0.01, 0.11) |  |  | 0.07 (0.04, 0.09) |  |
|  |  |  |  |  |  |  |
| Accent | CO (Chimdi Ota) | Reference | 0.65 |  | Reference | 0.01 |
|  | RO (Ruairi O’Kane) | -0.01 (-0.04, 0.03) |  |  | -0.05 (-0.09, -0.01) |  |
|  | RP (Riya Patel) | -0.01 (-0.03, 0.01) |  |  | -0.03 (-0.06, -0.01) |  |

b, unstandardised regression coefficient; CI, confidence interval; WER, word error rate

* Wald-type test for overall differences from quantile regression

With background-noise, a small significant advantage emerged for RO (-0.05 pp, 95% CI -0.09 to -0.01) and RP (-0.03 pp, 95% CI -0.06 to -0.01; overall P=0.001), but system and accent interaction remained non-significant (P=0.31) indicating that this modest accent effect was uniform

**Appendix Figure 1**

**Appendix Figure 1**	Average ROUGE metrics for all investigated ASR systems
